# The Role of Amino Acids in Tuberculosis Infection: A Literature Review

**DOI:** 10.3390/metabo12100933

**Published:** 2022-09-30

**Authors:** Fiki Amalia, Mas Rizky A. A. Syamsunarno, Rima Destya Triatin, Siti Nur Fatimah, Lidya Chaidir, Tri Hanggono Achmad

**Affiliations:** 1 Study Program of Medicine, Faculty of Medicine Universitas Padjadjaran, Bandung 40161, Jawa Barat, Indonesia; 2 Department of Biomedical Sciences, Faculty of Medicine Universitas Padjadjaran, Bandung 40161, Jawa Barat, Indonesia; 3 Center for Translational Biomarker Research, Universitas Padjadjaran, Bandung 40161, Jawa Barat, Indonesia; 4 Department of Public Health, Faculty of Medicine Universitas Padjadjaran, Bandung 40161, Jawa Barat, Indonesia

**Keywords:** amino acids, tuberculosis infections, host immune response, metabolomic

## Abstract

Recently, there was an abundance of studies being conducted on the metabolomic profiling of tuberculosis patients. Amino acids are critical metabolites for the immune system, as they might contribute to providing nutrients for the host intracellular pathway. In tuberculosis, several amino acids play important roles in both the mycobacteria infection mechanism and the host. Individual studies showed how the dynamics of metabolite products that result from interactions between *Mycobacterium tuberculosis* (*Mtb*) and the host play important roles in different stages of infection. In this review, we focus on the dynamics of amino-acid metabolism and identify the prominent roles of amino acids in the diagnostics and treatment of tuberculosis infection. Online resources, including PubMed, ScienceDirect, Scopus, and Clinical Key, were used to search for articles with combination keywords of amino acids and TB. The inclusion criteria were full-text articles in English published in the last 10 years. Most amino acids were decreased in patients with active TB compared with those with latent TB and healthy controls. However, some amino acids, including leucine, isoleucine, valine, phenylalanine, aspartate, and glutamate, were found to be at higher levels in TB patients. Additionally, the biomarkers of *Mtb* infection included the ratios of kynurenine to tryptophan, phenylalanine to histidine, and citrulline to arginine. Most amino acids were present at different levels in different stages of infection and disease progression. The search for additional roles played by those metabolomic biomarkers in each stage of infection might facilitate diagnostic tools for staging TB infection.

## 1. Introduction

In 2020, tuberculosis (TB) became the 13th major cause of death worldwide, and the main cause from a sole infectious pathogen before HIV/AIDS, which led to 1.3 million deaths. Approximately, ten million people in this world have been diagnosed with TB. Indonesia ranks second in the eight countries that contribute to two thirds of the total tuberculosis cases [1]. The global burden of tuberculosis cases in current years has forced us to study various characteristics of the disease.

The dynamics of metabolite products resulting from the interaction between *Mycobacterium tuberculosis* (*Mtb*) and the host play important roles in different stages of infection. Alterations in intracellular metabolism perform critical functions to stimulate and control the host defense mechanism. Immune cells further influence the cellular metabolism to adjust defenses against infectious cells and produce adequate energy to undergo host immunity functions. An abundance of evidence has confirmed the role of metabolism in the activation of immunity and host defenses against tuberculosis infection [2,3].

The amino acid is an organic compound that has a basic structure comprised of an amine group, carboxylic acid, and a side chain. The properties of the side chain further characterize the type of amino acid. Amino acids are basic building blocks in organisms. In addition to their role in protein synthesis, they are also an important nutrient for host immune cells. They assist the intracellular metabolic pathways of the host through ATP synthesis, nucleotide production, and redox balance. Activated growth factors and T cells stimulate the rapid proliferation of amino acids and increase amino-acid transporters [4]. Chronic infection is known to trigger malnutrition and further increase susceptibility to infectious diseases or other pathogens, resulting in a vicious cycle. The common metabolic pattern in infection includes hypermetabolism, increased nitrogen loss, increased gluconeogenesis, and increased fat oxidation. Thus, it is plausible to reduce protein loss with dietary provision therapy in patients with chronic infectious diseases [5]. In patients with TB, malnutrition may occur due to its symptoms, which include vomit, nausea, diarrhea, abdominal pain, and decreased appetite, all of which may decrease the patients’ dietary intake. In addition, the metabolic changes in the disease mechanism may also contribute to weight loss in these patients. According to the WHO, the recommended protein intake in patients with TB is approximately 15–30%, which is higher than the recommendation for a healthy person. Nutrient deficiencies can compromise the patients’ immune response and cause latent TB to convert to active TB [6].

Improvement in overall protein synthesis was shown after aromatic-amino-acid supplementation in infected acute malnourished infants, as it may have increased the leucine availability resulting from protein breakdown and for protein synthesis [7]. Glutamine supplements (20 g/day) given for 7 days to patients with HIV improved the levels of plasma GSH, glutamine, and glycine. In patients with HIV, the administration of N-acetylcysteine (NAC) resulted in significantly greater glutamine, cysteine, taurine, homocysteine, methionine, and GSH concentrations [8]. Studies related to amino-acid supplementation in patients with TB are still limited. Only one study evaluated the effects of L-arginine administration on TB, showing no benefits as adjunctive therapy [9].

Host immunity utilizes amino acids as the main energy source for cells including lymphocytes, fibroblasts, and enterocytes. Arginine and glutamine may activate TLR signaling, secretory immunoglobulin A (SIgA), and Paneth antimicrobials, as well as intestinal cell signaling pathways (NF-κB, MAPK, and PI3K–Akt) in host innate immunity. Serine is also needed for mediating T-cell function. Amino acids significantly affect the innate and adaptive host immune responses [10].

The number of metabolites was also found to be associated with an increase in cytokine concentration in the serum of patients with TB. A study conducted by Weiner et al. showed that amino acids significantly associated with some cytokines, such as IL-6, IP-10, and soluble interleukin 2 receptor alpha (sIL2ra) [5]. This relationship between amino acids and cytokines further emphasizes the role of amino acids in immunologic host response against *Mtb* infection [11].

Amino acids provide a nitrogen source for immune cells to combat *Mtb*, as well as nitrogen sources for *Mtb* metabolism. They might release nitric oxide, as it is known to have potent anti-mycobacterial properties, which can induce bacterial clearance [12]. It also provides ATP for host immune cells via the tricarboxylic acid cycle. More knowledge of the conceptual interaction between host and pathogen during *Mtb* infection might uncover those metabolomic changes, especially in amino acids, that might be beneficial for the host immune response against *Mtb* [3]. A previous study reviewed research on the targeting inhibitor for the amino-acid metabolism of *Mtb*, providing mostly in vitro-based studies [13]. Here, we review the role of the amino-acid metabolism pathway in Mtb infection based on clinical and in vivo studies to highlight the role of amino acids in tuberculosis infection. Considering the role of amino acids in biologic function and structure, as well as energy sources, the clinical understanding of the amino acids’ dynamic role is necessary to discover new biomarkers, diagnostic tools, and also the pathophysiologic process of tuberculosis infection.

Online resources including PubMed, ScienceDirect, Scopus, and Clinical Key were used to find articles with combination keywords of amino acids and TB. The inclusion criteria were full-text English articles published in the last 10 years. The exclusion criteria were literature reviews and articles that did not directly relate to our purpose after deep investigation. Searches were conducted using combinations of terms “tuberculosis” with either “metabolites” and “amino acids” or “phenylalanine”, “alanine”, “histidine”, “cysteine”, “threonine”, “glycine”, “glutamine”, “tryptophan”, “asparagine”, “valine”, “aspartate”, “glutamate”, “lysine”, “arginine”, “tyrosine”, “citrulline”, “methionine”, “leucine”, “isoleucine”, “serine”, “ornithine”, “aspartic acid”, or “ethanolamine”. Twelve articles were objectively selected as the most relevant for this review topic.

## 2. The Importance of Amino Acids in Tuberculosis Infection

### 2.1. Amino Acids Are Advantageous for Mycobacterium Tuberculosis

*Mtb* utilizes human nutrients as energy sources, and nitrogen is one of the crucial nutrients to its metabolism. The most important organic sources of nitrogen are amino acids. Amino acids have been identified to support *Mtb* intracellular growth. Amino acids go through deamination reactions to yield ammonium that serves as a nitrogen and carbon source to be used by *Mtb*. The amino acids involved in supplying nitrogen for *Mtb* growth are aspartate, glutamate, asparagine, and glutamine, which are found in human macrophages. Those amino acids are actively transported from the cytoplasm of human cells into the mycobacterium [14]. Based on Shin et al., other amino acids are produced by the mycobacterium itself, in the course of infection of the macrophage [15].

Instead of inorganic sources (e.g., ammonium and nitrate), amino acids serve as the main source of nitrogen for *Mtb* and include alanine, asparagine, aspartate, valine, glutamate, glutamine, ornithine, serine, arginine, isoleucine, and proline. They are involved in the survival mechanism of *Mtb* against intracellular stresses and its increased replication. *Mtb* growth has been shown to increase when using amino acids as the source of nitrogen compared with using ammonium. Glutamine is one of the primary sources of nitrogen used by *Mtb* [16]. Alanine, lysine, and glutamine significantly decrease in tuberculosis patients as they are quickly metabolized as nitrogen sources [17]. Another nitrogen source for *Mtb* is asparagine, which was shown to significantly decrease in the phagosome of active TB compared with the latent TB and the control groups [18]. The immunologic response to increased tryptophan being transformed into kynurenine by IDO1 in the setting of *Mtb* infection further confirms the complexity of this disease mechanism [19].

### 2.2. Amino Acids Are Beneficial to the Host Defense

Amino-acid metabolism has been reviewed as one of the important factors in host physiology for immunity, growth, and reproductive function. It provides an energy source for cells, as it is the primary compound involved in protein synthesis; it is a substrate for regulatory molecules, such as nitric oxide (NO), polyamines, and creatine, and a regulator of cell signaling pathways, as it is a target of the rapamycin complex 1 (mTORC1), MAPK, and NF-κB [19,20]. The activation of the host innate immune system may be mediated by amino acids (arginine and glutamine) that stimulate pathways related to secretory immunoglobulin A (SIgA), Paneth antimicrobials, TLR signaling, and intestinal cell signaling (NF-κB, MAPK, and PI3K–Akt) [21,22,23]. Glutamine activates T-cell-dependent and T-cell-independent pathways for SIgA secretion [24]. Leucine, glutamine, and gamma-aminobutyric acid have significant roles in mediating T-cell activation and differentiation, particularly for Th1 and Th17 cells [25,26]. Serine is also important for the optimalization of T-cell expansion. Thus, these studies are indicative of the critical role of amino acids in host innate and adaptive immune function [27].

**Figure 1:**
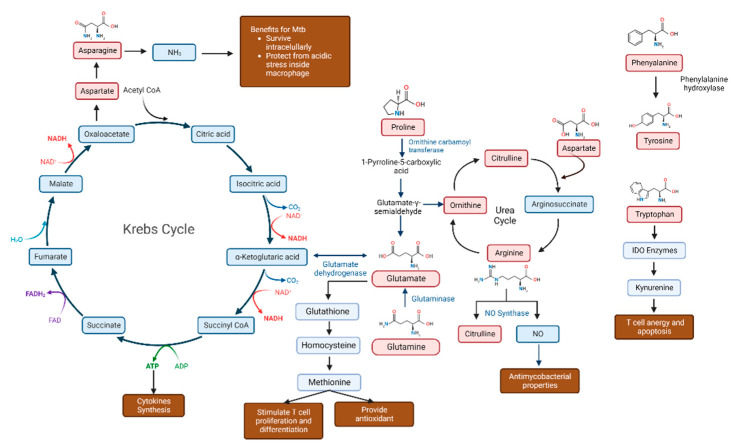
Pathways of amino acids roles in ATP and NO synthesis for *Mycobacterium tuberculosis* growth. Notes: Red boxes indicate amino acids; blue boxes show non-amino-acid metabolites; brown-colored boxes display amino-acid involvement in tuberculosis infection.

In a chronic wasting state of TB, amino acids diverge away for protein synthesis and lead to an oxidation process, causing body protein pool loss, termed “anabolic block” [28]. Nitric oxide, an extremely toxic molecule against a lot of compounds, is the dominant nitrogen source for immune cells involved in the host innate immune response against *Mtb*. It is produced by nitric oxide synthase (iNOS), which is expressed after inflammatory cytokine release. Interferon-γ (IFNγ) induces the transformation of amino acids, such as L-arginine, into citrulline and nitric oxide [29]. Several amino acids are beneficial to the host immune defense, as they are involved in the TCA cycle to synthesize ATP (Figure 1) [3].

### 2.3. Numerous Amino Acids Are Involved in Tuberculosis Infection

Studies addressing the metabolomic profile were conducted in many areas of the world, including South Africa, Indonesia, and Georgia. The majority of that research showed similar results regarding metabolomic profiles in patients with TB compared to either patients with latent TB or healthy control groups. A study from South Africa mentioned that histidine, cysteine, glutamine, tryptophan, and citrulline significantly declined in patients with TB. Lower levels of citrulline, and ornithine were also found in patients with active TB than in controls and then gradually increased to normal levels during therapy. Meanwhile, phenylalanine was raised in patients with TB and decreased after treatment [5]. These findings were identical to those of the studies by Weiner et al., Vrieling et al., and Ding et al. [11,30,31] (Table 1).

Elevated concentrations of branched-chain amino acids, including leucine, isoleucine, valine, and phenylalanine, were found in a group with TB infection. These findings were parallel between animal models and human studies. Several non-essential amines, such as aspartate and glutamate, were elevated in the serum of patients with TB. Methionine, threonine, asparagine, glycine, serine, and alanine were reduced in the same study. Distinct results were found for arginine, aspartate, lysine, and glutamate in research that we reviewed. Predominantly, arginine was raised in patients with TB as compared with the control group [11,30,39]. Hypoxia caused by granulomatous inflammation in the lungs due to tuberculosis infection may also explain the increase in several metabolites (serine) and decrease in others (cysteine and methionine) [37].

Biomarkers were developed from a metabolomic profile study in patients with TB. L-histidine was judged to be a biomarker in TB cases [17]. The citrulline-to-arginine ratio was a good predictor of *Mtb* infection. As phenylalanine raised in patients with TB, the ratio of phenylalanine to histidine was highlighted as biomarker for TB. The ratio between kynurenine and tryptophan was also higher in patients with active TB than in those with latent TB and controls [11,30].

### 2.4. Diverse Roles of Amino Acids in Tuberculosis Infection

#### 2.4.1. Tryptophan

A study conducted by Collins et al. showed that tryptophan and kynurenine were extremely different from other metabolites in patients with TB and controls and were highly altered after treatment. In the plasma of patients with TB, tryptophan was lower than in those with latent TB [4] and the control group and increased gradually after effective TB treatment. On the contrary, kynurenine plasma levels were higher in active TB disease and decreased gradually following effective TB management. The ratio of kynurenine to tryptophan also showed the same proportion as mentioned above [2]. Tryptophan was also significantly lower in the CSF of patients with TB meningitis than controls [40].

**Figure 2:**
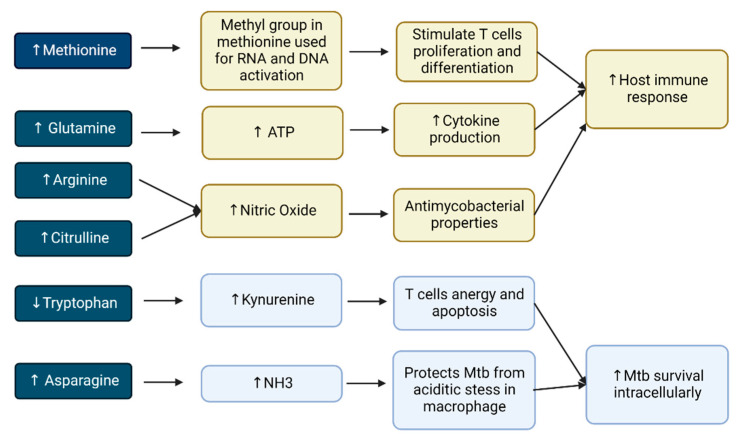
Diverse roles of amino acids in tuberculosis infection (light-blue color, amino-acid mechanisms to increase *Mtb* survival intracellularly; yellow color, amino-acid mechanisms increase host immune response).

Tryptophan, as well as other amino acids, has a two-sided role in the mechanism of TB, including being beneficial to either *Mtb* or host response. Its immunomodulatory effect linked with the induction of indoleamine 2, 3-dioxygenase 1 (IDO) enzymes benefits *Mtb* because decreased tryptophan may induce a low proliferation of CD4^+^ T cells. This is also related to the diminished number of *Mtb* antigen-specific T cells and inhibited Th17 cells. In addition, *Mtb* has the ability to create tryptophan itself, which induces human IDO activity to produce kynurenine, using the same pathway against host immunity [41,42]. Therefore, this mechanism enables *Mtb* to survive intracellularly, and causes immune tolerance to bacterial persistence. Therapeutics to inhibit IDO showed benefits to treat the disease. Moreover, tryptophan may maintain the host inflammatory response at the necessary level rather than causing intense inflammation, which is usually destructive (Figure 2) [2]. Blockage against the production of tryptophan by *Mtb* may result in a greater benefit to the host defense mechanism [43].

#### 2.4.2. Glutamine

The metabolism of glutamine is important during active infection for the host defense mechanism. Glutamine is involved in the generation of ATP through glutaminolysis. The enzyme glutaminase transforms it into glutamate, which is followed by conversion into α-ketoglutarate using glutamate dehydrogenase. Then, this can join the tricarboxylic acid cycle and produce ATP. Glutaminolysis is also necessary to induce innate immune memory in monocytes [44,45]. Infected macrophages may express individually distinct glutamine pathway genes for each person infected with tuberculosis. A decrease in the levels of interleukin 1β, interferon γ, and interleukin 17 in the peripheral blood mononuclear cells of the host is provoked by *Mtb* in medium without glutamine. Specific inhibitors against glutamine metabolism result in a lower synthesis of cytokines, including IFN-γ, IL-17, and IL-22. These T-cell-derived cytokines play a key role in the immunity against *Mtb,* which may influence risk factors of infection and the course of the disease. Consequently, glutamine metabolism may modulate the host innate immunity against infection by *Mtb* (Figure 2) [3].

#### 2.4.3. Asparagine

Aspartate is not the primary nitrogen source for *Mtb*, as the gene directing aspartate transport (AnsP1) is not required for intracellular survival, although it was found to be important for nitrogen production in a murine model of TB [29,46]. Asparagine supports nitrogen sources for mycobacterial growth through releasing ammonia and the maintenance of pH [47]. It is found intracellularly in macrophages, which denotes that asparagine can be easily obtained by *Mtb* throughout infection in vivo. The bacillus scavenges it for assimilation induced by asparaginase. This enzyme hydrolyzes asparagine into aspartate and ammonia, and these nitrogen sources enable microorganisms to resist human immune response, protected from acidic stress inside macrophages (Figure 2) [16,48].

#### 2.4.4. Arginine

Arginine metabolism in the setting of infection by *Mtb* occurs in activated myeloid cells. Macrophages release inducible nitric oxide synthase (iNOS) to produce high levels of nitric oxide (NO), which have antimicrobial properties, especially against mycobacterial infection. Research using transgenic mice with deficiency in Nos2, which encodes iNOS, showed increased vulnerability to infection by *Mtb* (Figure 2) [12].

L-arginine supplementation in patients with TB increased NO synthesis, which helped the immune response against *Mtb*. The result of this study showed insignificant benefits, including enhanced sputum clearance, and alleviated symptoms, such as cough and chest pain, in patients with TB and without HIV [9]. Another study showed no clinical advantages of oral supplementation with L-arginine in patients with TB, which may have been due to an inadequate availability for the host immune response caused by ingestion in the enterocytes or through import into liver hepatocytes [49].

#### 2.4.5. Phenylalanine and Tyrosine

The metabolic products of these amino acids (e.g., norepinephrine, gentisic acid, 4-hydroxybenzoic acid, hydroquinone, and 4-hydroxyhippuric acid) were increased in patients with active TB compared with controls [50]. Phenyllatic acid and phenylacetic acids were also elevated in the urine of patients with TB, which implied the accumulation of phenylalanine. The metabolism of phenylalanine relies on phenyalanine hydroxylase (PAH) to convert it into tyrosine helped by cofactor tetrahydrobiopterin (BH4). When a deficiency in these enzymes occurs, the accumulation of phenylalanine causes transamination to produce phenylpyruvic acid, which undergoes either oxidation into phenylacetic acid or reduction to phenyllactic acid [34]. On the other hand, a study presented that essential amino acids and semi-essential amino acids deregulated in active TB include phenylalanine and tyrosine, respectively [50]. The serum levels of these amino acids depend on dietary protein and its uptake from the portal circulation system. In patients with active TB and wasting syndrome, these amino acids can be liberated from tissue storage or circulation proteins [34].

#### 2.4.6. Citrulline

Citrulline was firstly studied in watermelon juice (Citrullus vulgaris) by Mandel et al. in 2005 [51]. It can be obtained from diet or from metabolic changes in arginine or ornithine via NO synthases or ornithine carbamoyl transferase, respectively [52]. This type of amine has potent antimycobacterial effects by supplying nitrogen to murine macrophages and T cells when arginine is low. Citrulline is an important intermediary of the urea cycle, whose formation via ornithine and ammonia initiates the cycle, resulting in aspartic acid, via the transformation of arginosuccinate from arginine. The antimycobacterial effect of citrulline manifests via an alternative intracellular arginine [53,54]. This pathway plays a role in immune protection during infection with *Mtb*, when citrulline becomes extremely important. Lower levels of this amino acid might be harmful for patients with TB (Figure 2) [30].

#### 2.4.7. Methionine

During infection, the production of DNA, proteins, and other biomolecules increases to enable cell multiplication and the rapid expansion of T cells but might cause metabolism overload. The full activation of T cells requires methionine import and the rapid upregulation of Slc7a5. The methyl group from methionine is used in the biochemical reactions of RNA and DNA that stimulate the proliferation and differentiation of T cells. Methionine, an essential amino acid, plays a crucial role in this period of infection [55].

Methionine contains sulfur that can be oxidized. This alters methionine into methionine sulfoxide. Then, the enzyme methionine sulfoxide reductase may reduce sulfoxide to the original form of methionine. Methionine residues provide efficient antioxidant defenses through reaction with oxidized species (Figure 2) [56]. High levels of methionine in patients with TB are regarded as markers of oxidative stress, due to its susceptibility to be oxidized by reactive oxygen species (ROS). During antibiotic therapy, methionine levels decreased in patients with TB [30].

### 2.5. Amino-Acid Profile in Multidrug-Resistant TB

A study conducted by Rego et al. measured the metabolite levels in multidrug resistant (MDR), extensively drug resistant (XDR), and drug sensitive (DS) TB. Proline, isoleucine, and hercynine significantly differed among those groups. Proline significantly increased in DS compared with MDR and XDR. The lowest levels of isoleucine were in the MDR group compared with DS. Hercynine was higher in the XDR group compared with MDR and DS. Elevated hercynine was caused by an increased production of ergothioneine to combat oxidative stress due to drug-resistance mutations situated in a gene important for the survival of *Mtb*. These amino acids were also involved in a pathway of phenotypic drug resistance, which might have impaired ergothioneine synthesis and increased susceptibility to rifampicin isoniazid, bedaquiline, and clofazimine [57].

### 2.6. Targeting Amino Acids for Tuberculosis Drug Development

Since the amino-acid role in tuberculosis was confirmed by studies, ongoing research has been dedicated to evaluating drugs targeting amino acids to combat this disease. Consalvi et al. provided an interesting review of tryptophan inhibitor biosynthesis for the treatment of tuberculosis. Fluoro-anthranilates, including 2-amino-6- fluorobenzoic acid (6-FABA), 5-FABA, and 4-FABA, contributed to inhibit *Mtb* growth in mice through fluorinated tryptophan synthesis. However, the results of treatment using tryptophan biosynthesis were still insignificant, as they included a decline in colony forming units (CFUs) and low antimicrobial effect, and need to be upgraded [58,59]. Another study reported the supplementation of L-arginine in pulmonary tuberculosis patients at a 6.0 g dosage per day for 8 weeks as adjunctive therapy and showed clinically and microbiologically non-significant outcomes. This study suggested higher doses and more sample participants [9]. Further studies are still needed to upgrade the targeting of amino acids for tuberculosis drug discovery.

## 3. Conclusions

Patients with TB show decreases in the levels of the majority of amino acids compared with healthy controls or those with latent TB. This is associated with active disease, as they become nitrogen sources for host immune response against *Mtb* and are also utilized by *Mtb* to continue its growth. Several amines are normalized during antibiotic therapy. Studies conducted on metabolomic profiling have found that the biomarkers of *Mtb* infection include the ratios of kynurenine to tryptophan, phenylalanine to histidine, and citrulline to arginine. Knowledge of additional roles for these metabolomic biomarkers in each stage of the disease might facilitate the diagnose and staging of TB infection. How the levels of essential dietary, semi-essential, and non-essential amino acids contribute to increase host immunity and prevent wasting syndrome in patients with TB is not yet known and requires further investigations.

## Figures and Tables

**Table 1:** Amino-acid levels in clinical and in vivo studies.

Authors	Subject	Proper-ties	A	P	H	C	Th	G	Gl	As	Tr	V	At	Glt	L	Ty	M	Ci	O	Ar	S	Le	Iso	E
**Weiner et al., 2012** [5]	44 TB 46 latent TB 46 HC	UPLC-MS/MS GC-MS			↓	↓	↓		↓		↓		↓											
**Weiner et al., 2018** [11]	4462 progressors *	GC-MS			↓				↓															
**Albors-Vaquer., 2020** [17]	35 HC 15 active TB 30 household contacts	^1^H-NMR	↓												↓									
**Zhou et al., 2013** [32]	38 TB 39 HC	^1^H-NMR spectroscopy	↓	↑				↓	↑					↑	↑	↑								
**Frediani et al., 2014** [33]	17 TB	NMR											↑	↑										
**Cho et al., 2020** [18]	21 active TB 20 latent TB 38 HC								↓	↓	=		↑				↓							
**Vrieling et al., 2019** [30]	48 TB 20 TB-DM 48 HC	LC-MS/MS		↑	↓ ↓↓		↓ ↓↓	↓ ↓↓	↓ ↓↓		↓ ↓↓		↑	↓ ↓↓				↓ ↓↓	↓ ↓↓	↑	↓ ↓↓			
**Luier and Loots, 2016** [34]	46 active TB 30 HC	URINE-MS									↓					↑								
**Yi et al., 2019** [35]	35 HC 35 untreated TB 31 two-month treated TB subjects 29 cured TB subjects	MS		↑	↓						↓					↑				↓				
**Huang et al., 2019** [36]	35 TB 35 controls 35 CAP 31 LC	LC-MS/MS												↓										
**Conde et al., 2022** [37]	37 PTB 12 EPTB	^1^H-NMR				↓	↓			↓		↓	↑	↑			↓		↑		↑			
**Magdalena et al., 2022** [38]	15 TB children 52 LTBI 20 NMP 149 HC	LC-MS/MS							↓						↑			↓						
**Shin et al., 2011** [15]	Mice #	^1^H-NMR		↑								↑										↑	↑	
Ding et al. **, 2020 [31]	20 TB 20 HC zebrafish mice	LC-MS NMR spectroscopy		↓		↓	↓			↓	↓						↓	↓			↓	↓		↓

Abbreviations: HC, healthy controls; MS, mass spectrometry; NMR, nuclear magnetic resonance; UPLC-MS, ultra-performance liquid chromatography–mass spectrometry; GC-MS, gas chromatography–mass spectrometry; LC-MS, liquid chromatography–mass spectrometry. A, Alanine; P, Phenyalalanine; H, Histidine; C, Cysteine; T, Theonine; G, Glycine; Gl, Glutamine; As, Asparagine; Tr, Tryptophan; V, Valine; At, Aspartate; Glt, Glutamate, L, Lysine; Ty, Tyrosine; M, Methionine; Ci, Citrulline; O, Ornithine; Ar, Arginine; S, Serine; Leu, Leucine; I, Isoleucine; E, Ethanolamine. ↑ Higher in TB patients compared with other groups. ↓ Lower in TB patients than in other groups. ↓↓ Lower in TB-DM patients than in TB. = no significant differences between TB and other groups. * TB progressors: close contact to TB patients and developing TB within 2 years of observation. ** These amino acids were decreased in TB patients, zebrafish larvae models, and mice models. # Mice models had increased levels of amino acids in the serum, lung, spleen, and liver tissues.
